# The feasibility of field collected pig oronasal secretions as specimens for the virologic surveillance of Japanese encephalitis virus

**DOI:** 10.1371/journal.pntd.0009977

**Published:** 2021-12-03

**Authors:** Shyan-Song Chiou, Jo-Mei Chen, Yi-Ying Chen, Min-Yuan Chia, Yi-Chin Fan

**Affiliations:** 1 Graduate Institute of Microbiology and Public Health, College of Veterinary Medicine, National Chung Hsing University, Taichung, Taiwan; 2 Department of Veterinary Medicine, College of Veterinary Medicine, National Chung Hsing University, Taichung, Taiwan; 3 Institute of Epidemiology and Preventive Medicine, College of Public Health, National Taiwan University, Taipei, Taiwan; WRAIR, UNITED STATES

## Abstract

Virologic surveillance of Japanese encephalitis virus (JEV) relies on collecting pig blood specimens and adult mosquitoes in the past. Viral RNAs extracted from pig blood specimens suffer from low detecting positivity by reverse transcription PCR (RT-PCR). The oronasal transmission of the virus has been demonstrated in experimentally infected pigs. This observation suggested oronasal specimens could be useful source in the virus surveillance. However, the role of this unusual route of transmission remains unproven in the operational pig farm. In this study, we explore the feasibility of using pig oronasal secretions collected by chewing ropes to improve the positivity of detection in commercial pig farms. The multiplex genotype-specific RT-PCR was used in this study to determine and compare the positivity of detecting JEV viral RNAs in pig’s oronasal secretions and blood specimens, and the primary mosquito vector. Oronasal specimens had the overall positive rate of 6.0% (95% CI 1.3%–16.6%) (3/50) to 10.0% (95% CI 2.1%–26.5%) (3/30) for JEV during transmission period despite the negative results of all blood-derived specimens (*n* = 2442). Interestingly, pig oronasal secretions and female *Culex tritaeniorhynchus* mosquito samples collected from the same pig farm showed similar viral RNA positive rates, 10.0% (95% CI 2.1%–26.5%) (3/30) and 8.9% (95% CI 2.5%–21.2%) (4/45), respectively (*p*> 0.05). Pig oronasal secretion-based surveillance revealed the seasonality of viral activity and identified closely related genotype I virus derived from the mosquito isolates. This finding indicates oronasal secretion-based RT-PCR assay can be a non-invasive, alternative method of implementing JEV surveillance in the epidemic area prior to the circulation of virus-positive mosquitoes.

## Author summary

Mosquito-borne Japanese encephalitis virus (JEV) has either endemic or seasonal patterns of transmission in Asia and Australia. Most hosts infected by the virus remains asymptomatic but can result in severe encephalitis in humans and horses, and abortion or stillbirth in pregnant sows. Isolation of virus in adult mosquitoes or pig seroconversion has been used as an early indicator of upcoming JE outbreak in humans. Genotype identification of the virus is important since current human and domestic animal vaccines are all genotype III (GIII) specific. GIII vaccine elicited immunity has reduced cross-protections to genotypes other than GIII. Our virologic surveillance using pig’s oronasal secretion detected higher prevalence and earlier genotype I virus activity than using pig’s blood and mosquitoes, respectively. This proposed surveillance tool might be more effective that will allow the public health agency to properly implement the preventive measures, such as implementing mosquito control, encouraging booster vaccination, and encouraging the use of mosquito repellent, to reduce the impact of upcoming outbreak. Collection of pig’s oronasal secretion is non-invasive to pigs and less technically demanding to operators. Thus we propose the use of pig’s oronasal secretions as the novel source of specimens for virologic surveillance to replace the traditional pig blood or adult mosquito specimens to monitor and control JE outbreak/epidemic in the future.

## Introduction

Japanese encephalitis virus (JEV) is a zoonotic mosquito-borne flavivirus. Most infection by the virus remains asymptomatic but can result in severe encephalitis in dead-end hosts, such as humans and horses, and abortion or stillbirth in pregnant sows [1]. An annual 68,000 JE human cases is estimated with 20–30% case-fatality and 30–50% of survivors suffer neurological sequelae [2]. Currently there is no specific treatment for JE. Vaccination is considered as the most effective strategy to reduce JE burden in humans and sows [2,3].

JEV can either circulate year-round in tropic regions or cause seasonal outbreaks in temperate regions of Asia and Australia [4]. Potential expansion of the virus to Africa and Italy had been reported recently [5–7]. The virus circulates primarily between pigs and *Culex tritaeniorhynchus* mosquitoes in June-July, and the circulation decreases after spread to humans and horses [1]. Porcine JEV infection rate associated with human cases was generally accepted for the epidemic regions of Asia [8,9]. Classic surveillance determines seroconversion or seroprevalence in pigs to monitor the virus activity in epidemic or endemic areas [10,11]. The human outbreaks usually start at 1 to 2 months after 50% of pigs seroconverted [10,12]. Because of the short duration of viremia in pigs, viremia or viral RNA positivity rates are rarely used [13,14]. Thus, mosquito samples are more frequently collected than pig blood specimens for the virologic surveillance [15]. JEV-positive mosquitoes appeared earlier or the same month as the first reported human case [12,16]. Therefore, current surveillance uses pig seroconversion or JEV-positive mosquitoes as an early indicator for the coming epidemic season in human [1].

Phylogeny classifies JEV isolates into five genotypes. Each genotypes have an unique and sometime overlapping geographical distribution [4]. Phylogenetic analysis of the viruses demonstrates the replacement of genotype III (GIII) virus by genotype I (GI) virus, and illustrates geographical expansion of the emerging GI viruses in the epidemic regions of Asia [17,18]. However, GII, GIII, and GIV viruses remain circulating in Singapore, Philippine, and Indonesia, respectively [19,20]. Emerging GI virus has been showed it is capable of enhancing replication in experimentally infected pigs and is less susceptible to the current GIII vaccine-induced antibodies [21]. This suggests molecular surveillance and genotype tracking in pigs are critical to provide the important information of controlling JEV. Pig-based seroprevalence studies remain essential for the area like Hokkaido in Japan, where high seroprevalence is observed in pigs without the evidence of circulating JEV-positive mosquitoes [22].

Experimentally infected pigs demonstrate that JEV can persist in pig tonsils for 25 days and transmit to naïve co-inhabitants via oronasal route longer than the duration of viremia [13,23–26]. These studies suggest that the potential use of pig oronasal specimens for JEV surveillance. The longer period of virus persistence might overcome the issue of short viremia in pig blood-based surveillance. However, the role of this unusual route of transmission remains unproven in the farm-raised pigs. This study aims to evaluate and compare the performance of molecular surveillance using pig oronasal secretions, pig blood specimens and mosquito samples.

## Methods

### Ethics statement

We collected pigs’ oronasal samples from a farrow-to-finish farm, which is semi-open with natural ventilation and surrounded by rice paddies. This field study protocol was approved by Institutional Animal Care and Use Committee in National Taiwan University College of Medicine and College of Public Health (Protocol No: 20180260).

### Sample collection and processing

Reproductive sows receive annual JE vaccination in Taiwan, thus we collected blood and oronasal specimens from piglets older than 12-week old with undetectable JEV specific maternal antibodies based on our previous study [27]. Blood specimens were collected from an operational pig farm in 2009 or pig slaughterhouse in 2010 and 2014 in Taichung. Plasma and peripheral blood mononuclear cells (PBMC) were recovered from the blood samples contained a final concentration of 0.33% sodium citrate (Sigma-Aldrich) after centrifugation at 3,000 rpm for 15 minutes. From April 2018 to April 2020, we collected oronasal secretions of older than 12-week non-vaccinated piglets [27], and female *Culex tritaeniorhynchus* mosquitoes from the operational pig farm in Taichung. We prepared 50 cm of bleach-free cotton ropes (2 cm diameter) in autoclavable plastic bags. The ropes were dried and cooled at room temperature after sterilization in the autoclave. We provided one chewable sterile cotton rope per 8 to10 pigs per pen for 10–15 minutes chewing. We harvested oronasal secretion from chewed cotton ropes by wringing the ropes and collected the supernatant after centrifugation at 4,500 rpm for 10 minutes at 4°C. Pig’s oronasal secretions were collected monthly from 80 to 100 pigs in different holding pens. We collected mosquitoes once per month coincident with oronasal sample collection, from May-2018 to November-2018, May-2019 to July-2019, and September-2019 to January-2020. Mosquitoes within the vicinity of pig farm were captured from 5:00 PM to 8:00 PM by light-trap, and 50 female *Culex tritaeniorhynchus* mosquitoes were pooled into one tube. All the specimens were stored in −80°C freezer until used.

### Multiplex genotype-specific reverse transcription PCR (RT-PCR)

We extracted viral RNA and performed RT-PCR as previously described [28,29]. Briefly, viral RNA was extracted from plasma, PBMC and the supernatant of pig’s oronasal secretions, and homogenised *Culex tritaeniorhynchus* mosquitoes by using a Viral RNA Extraction kit (Viogene) and following a protocol provided by the manufacturer. The recovered viral RNA was reverse transcribed into random hexamers primed cDNA using Superscript III reverse transcriptase (Invitrogen). JEV multiplex PCR was carried out in GoTaq Master Mix (Promega) with GI-specific, GIII-specific, and JEV universal primers [29].

### Phylogenetic analysis

We amplified and sequenced the virus envelope (E) gene with JEV primers as previously described [28]. Full nucleotide sequence of the envelope protein (1,500 nt) was used in the phylogenetic analysis. The phylogenetic tree was inferred by using the maximum likelihood method and general time reversible model. The reliability of tree topology was assessed with 1,000 bootstrap replicates. This evolutionary analyses were carried out in MEGA X [30].

### Statistics

The positive rates of oronasal secretions and mosquitoes for JEV and binomial exact 95% confidence interval (CI) were calculated and compared by using Fisher exact test with R version 4.0.5 (https://www.R-project.org/).

## Results

### Detection of JEV RNA in farm-raised pigs’ plasma and PBMC using multiplex RT-PCR

We carried out a longitudinal study to monitor JEV infection in 18 piglets raised in the operational pig farm at two-week interval during March to August 2009. A total of 154 and 72 plasma or PBMC specimens were respectively collected during JE transmission and non-transmission season (Table 1). We tested all the specimens with multiplex genotype-specific RT-PCR [29] and found negative results for all. However, these pigs were seroconverted in June, 2009 (S1 Fig) and a part of this observation was described in the previous study when JEV-positive mosquitoes were identified in the same farm [28]. To increase the possibility of identifying viremic pigs, we collected blood specimens in pig slaughterhouses in Taichung, Taiwan [10]. A total of 550 plasma and 1390 PBMC samples were collected from one pig slaughterhouse in 2010 and 2014. Again, we found all the collected plasma and PBMC specimens were negative by the RT-PCR regardless of when the collection was made in JEV transmission or non-transmission season (Table 1). Our previous study identified JEV-positive mosquitoes in the pig farm located in the same district as the pig slaughterhouses in 2010 [28]. These results indicated plasma- and PBMC-based RT-PCR had low positivity for detection of JEV infection in pigs.

**Table 1:** Detection of JEV in pig plasma and PBMC specimens collected from a pig farm and a pig slaughterhouse in Taichung city, Taiwan.

Year	Places	Specimens	Collection periods^*a*^	Number of samples	RT-PCR
2009	Farm	Plasma	Transmission	154	0
			Non-transmission	72	0
2009	Farm	PBMC	Transmission	154	0
			Non-transmission	72	0
2010	Slaughterhouse	Plasma	Transmission	300	0
			Non-transmission	0	0
2010	Slaughterhouse	PBMC	Transmission	300	0
			Non-transmission	0	0
2014	Slaughterhouse	Plasma	Transmission	0	0
			Non-transmission	250	0
2014	Slaughterhouse	PBMC	Transmission	843	0
			Non-transmission	247	0

^*a*^JEV transmission season is from May to October and the non-transmission season is the remaining months.

### Comparison between field-collected oronasal secretions and blood samples as the source for detection of JEV RNA in pigs

**Fig 1:**
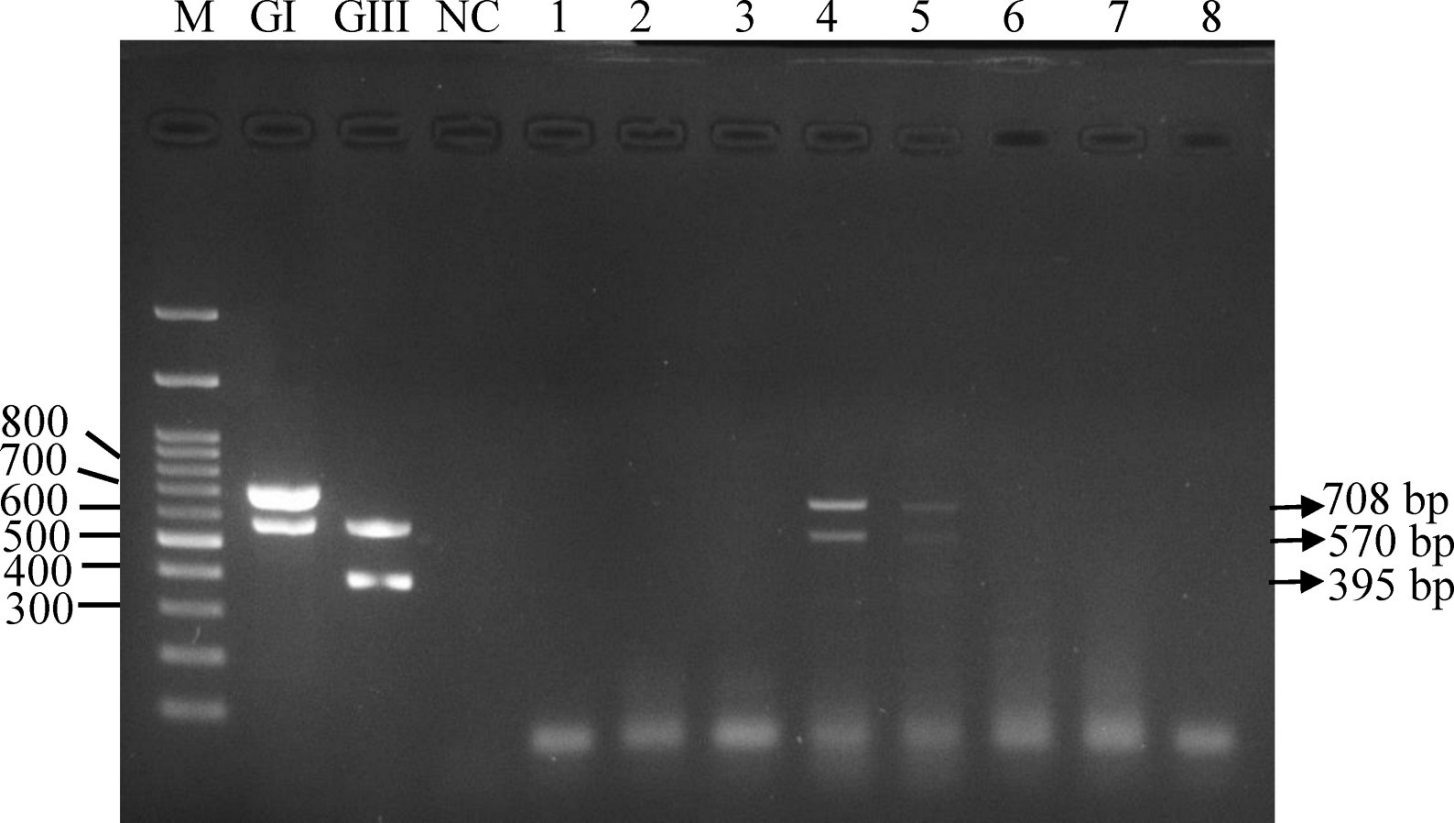
Detection of JEV in pig’s oronasal secretions by multiplex genotype-specific RT-PCR. Eight oronasal secretions (1–8), genotype I (GI) and III (GIII) virus control, and one negative control sample (NC) were presented on the electrophoresis gel. The size of universal, GI-, and GIII-specific PCR products are 570 bp, 708 bp, and 395 bp, respectively.

Previous studies demonstrated oronasal shedding of JEV in experimentally infected pigs [13,23]. These studies suggested the potential of pig oronasal secretions can be used for JEV surveillance. We first verified this potential by performing multiplex JEV genotype-specific RT-PCR with pig oronasal secretions collected from the operational farm. JEV RT-PCR was able to amplify GI-specific product from two oronasal samples collected in the transmission season (Fig 1). We subsequently compared the performance and positivity of RT-PCR using oronasal secretion and blood specimens in pig farms, Taichung, 2018. We observed 6.0% (95% CI 1.3%–16.6%) (3/50) of oronasal samples were positive for the virus in contrast to all negative results in blood samples (Table 2). These results suggest RT-PCR is more sensitive using oronasal secretions than blood specimens in farm-raising pigs.

**Table 2:** Detection of JEV in pig oronasal secretions and plasma collected from pig farms, Taichung city, Taiwan, 2018.

Specimens	Number of samples	RT-PCR
Plasma	50	0
Oronasal secretion	50	3

### Comparison between field-collected oronasal secretions and mosquito pools to determine seasonality of JEV in Taiwan

*Culex tritaeniorhynchus* mosquitoes are the primary vector as well as the most common mosquitoes in pig farms [16]. JEV-specific RT-PCR is the most common approach for detecting viral RNAs and to determining the seasonality of JEV. Table 2 suggests oronasal secretions were better source of viral RNAs than pig bloods for RT-PCR based molecular surveillance. Therefore, we further compared the positivity and performance of molecular detection using oronasal secretions and female *Culex tritaeniorhynchus* mosquito pools to determining the seasonality of JEV in the operational pig farms (Table 3). During JE transmission months, GI virus was first been detected in pig oronasal secretions in May without the evidence of the presence of circulating JEV-positive mosquitoes. By June, the virus was presented in pig oronasal secretions and mosquito pools. The overall positivity rate was 10.0% (95% CI 2.1%–26.5%) in pig oronasal secretions as comparable to 8.9% (95% CI 2.5%–21.2%) (*p*> 0.05) in mosquito pools. Virologic surveillance using pig oronasal secretions may detect JEV activity earlier than using mosquito specimens.

**Table 3:** Detection of JEV in pig oronasal secretions and female *Culex tritaeniorhynchus* mosquito pools collected from a pig farm, Taichung city, Taiwan, 2019.

Specimens	Collection periods	Months	Number of samples	RT-PCR	Positive rate (95% CI) (%)
Oronasal secretions	Transmission	May	10	2	10.0 (2.1–26.5) (3/30)
		Jun.	10	1	
		Jul.	10	0	
	Non-transmission	Nov.	10	0	0 (0.0–11.6) (0/30)
		Dec.	10	0	
		Jan.	10	0	
Mosquitoes	Transmission	May	15	0	8.9 (2.5–21.2) (4/45)
		Jun.	21	4	
		Jul.	9	0	
	Non-transmission	Nov.	3	0	0 (0.0–41.0) (0/7)
		Dec.	2	0	
		Jan.	2	0	

### Seasonality of JEV detected in oronasal secretions collected from the pig farm

**Fig 2:**
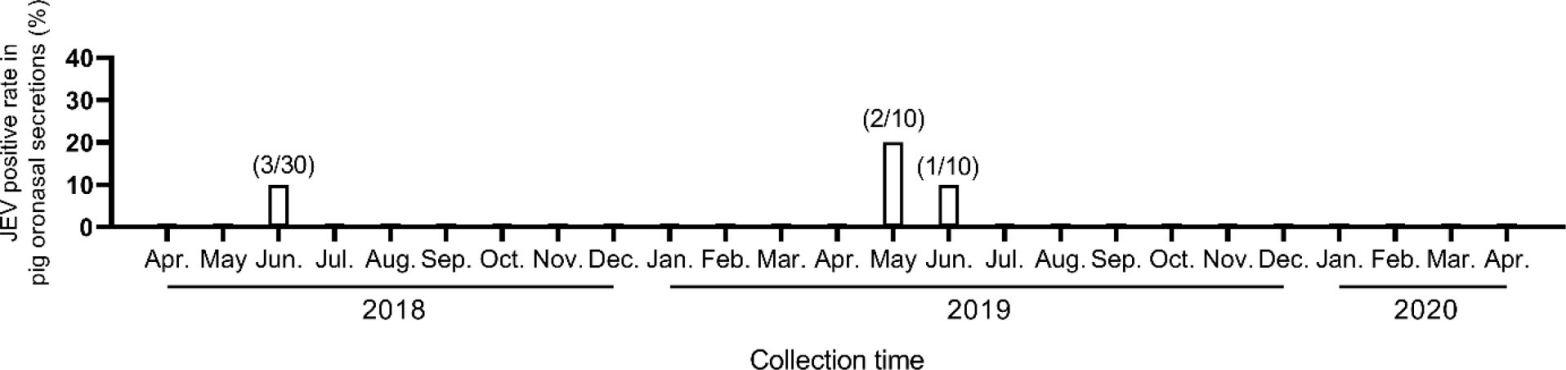
Seasonality of JEV-positive pig oronasal secretions in a pig rearing farm, Taiwan, 2018–2020. The positive rate is calculated by dividing the numbers of JEV-positive pen (numerator) by the total number of pen subjected to specimen collection (denominator).

Traditional surveillance to forecast upcoming JE season is determined by the pig seroconversion and the viral-positive mosquito pool [1]. Chewable rope for collecting pig’s oronasal secretion is a non-invasive, efficient method. Here, we aimed to evaluate the potential of using this method to detecting viral RNAs by JEV genotype-specific RT-PCR to replace the traditional surveillance method. The oronasal secretions were collected monthly from the operational pig farm between April-2018 to April-2020 (Fig 2). During this 25-month period, pig’s oronasal secretions were viral RNA-positive in June-2018 [10% (95% CI 2.1%–26.5%), 3/30], May-2019 [20% (95% CI 2.5%–55.6%), 2/10] and June-2019 [10% (95% CI 0.3%–44.5%), 1/10]. Specimens collected from the remaining 22 months were viral RNA negative. This result indicated JEV season started in May or June of 2018 and 2019, respectively. Virus in pig’s oronasal secretions returned to undetectable level from July to the next April. This seasonality of JEV activity was also observed and coincident with pig seroconversion (S1 Fig).

### Phylogeny of JEV using pig’s oronasal secretions

**Fig 3:**
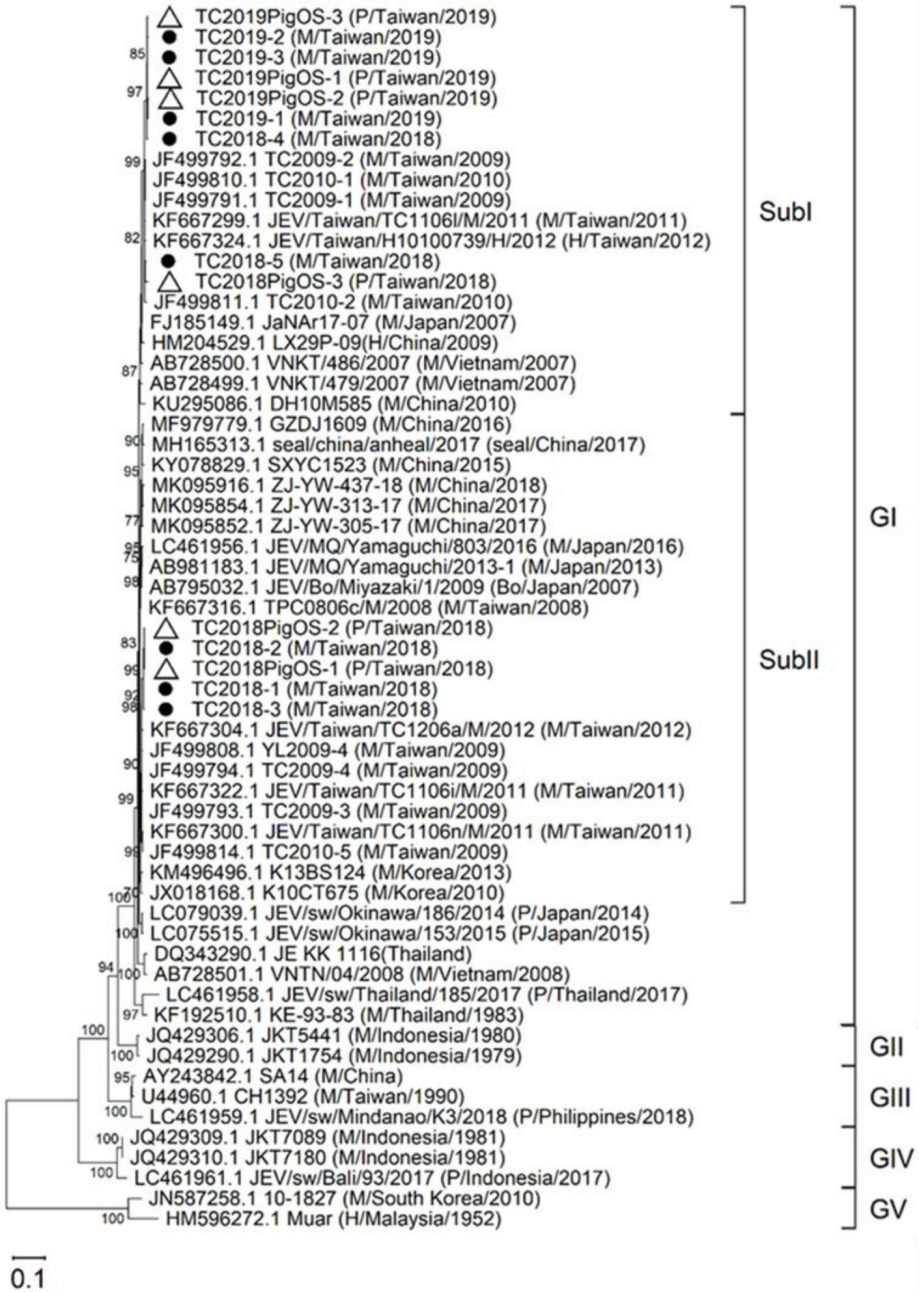
Maximum-likelihood tree of JEV detected in pig oronasal secretions (empty triangle) and mosquitoes (filled circle) in Taiwan, 2018–2019, and reference E gene for phylogeny. The bootstrap value (%) ≥70 is indicated on the node. Scale bar indicates the number of substitutions per site. The source, country, and year of isolation is noted in parentheses. M, mosquito; P, pig; H, human. GI-V, genotype I-V; SubI and SubII, subcluster I and II.

Phylogenetic analysis of pig blood-, mosquito-, and human-derived JEV suggests the utility of this analysis to determine the transmission cycle, the virus endemicity, and the genotype expansion [4,15,17,19]. In this study, we identified 14 viral RNA positive collections (6 from oronasal secretions and 8 from mosquito pools) (S1 Table). We amplified, sequenced and analysed the E protein sequence of all 14 viral positive specimens as previously described [28]. All 14 viral positive collections were classified and grouped into closely related GI subcluster I (subI) and II (subII) by the phylogenetic analysis (Figs 3 and S2). Close phylogenetic relation between oronasal secretion- and mosquito-derived sequences supports that the pig farm maintained GI virus circulation between pigs and mosquitoes. Viruses detected in this study are closer related to 2009–2012 Taiwanese isolates than the recent JEV isolates from other Asian countries. This evidence supports the endemicity of JEV in Taiwan.

## Discussion

The pig-mosquito-pig cycle is the primary mechanism to maintain the JEV circulation in the endemic and the epidemic regions of Asia and Australia (1). Emerging GI virus shows enhanced viral replication and reduced the sensitivity to vaccine-induced neutralizing antibodies in pigs [21,27]. Thus, it is important to monitor JEV-genotype circulation and evolution in pigs. However, JEV isolates are frequently identified from mosquitoes because of short viremia and higher seroprevalence in pigs, especially in JE endemic area [13–15,19,23]. Previous virologic surveillances using pig blood specimens reported the positive rates of 3.4% (1/29) in sentinel pigs and 0.5% (5/945) in randomly selected pigs [14,19]. These studies carried out short bleeding interval of 10 days or amplified the specimens in cells before proceeding to RT-PCR. Without prior amplification, our study indicates higher prevalence of GI virus in pig oronasal secretions [6% (95% CI 1.3%–16.6%) to 10% (95% CI 2.1%–26.5%) in 240 to 500 randomly selected pigs] than in pig blood samples [0% (95% CI 0%–2.4%) in 18 sentinel pigs and 0% (95% CI 0%–0.3%) in 1143 randomly selected pigs] during the viral transmission season. This observation is supported by longer period of the virus presented in oronasal shedding, up to 6 days than 2- to 3-day viremia in infected pigs [13,23]. We were able to detect the virus from either sero-positive or -negative oronasal secretions (S2 Table). This observation might be due to virus continue replicate in pig tonsils even after seroconversion [23], thus, suggests oronasal secretion might increase the positivity of detecting JEV in seropositive pigs in the endemic area [19]. Similarly, human throat swabs have a higher positivity of detecting JE viral RNAs than cerebrospinal fluid and serum specimens in JEV confirmed cases [31]. Viruses in the same genus, such as dengue and Zika viruses, can also be detected in human respiratory specimens [32,33]. As compared to collect blood specimens in pigs, the chewable rope method to collect oronasal secretions requires less technical demand and is less stressful for pigs.

Both pig oronasal secretions and mosquito surveillance revealed the same seasonality of JEV GI virus in the current as well as in the previous study in Taiwan [16]. We found the virus appears earlier in pig oronasal secretions than mosquitoes. The appearance of detecting JEV-positive oronasal secretion coincidenced with the first confirmed human case in 2018 and 2019 in Taiwan (obtained from the National Infectious Disease Statistic System, Centers for Disease Control-Taiwan; https://nidss.cdc.gov.tw/en/).

Genotype identification is important for the areas where two or more genotypes are co-circulated since current human and domestic animal vaccines are all GIII specific [34–37]. Immunity elicited by GIII vaccines is less protective to genotypes other than GIII [38]. Phylogeny can also be used to monitor virus endemicity, introduction, and expansion [5,19,39]. Our data showed that the pig oronasal secretion provided the similar virologic information as the mosquito collected in the vicinity of pig farm [16]. Our studies also showed that oronasal secretion and mosquito viruses are phylogenetic close related GI viruses after the occurrence of genotype replacement in Taiwan [16,28,40]. Mosquito surveillance requires tedious efforts to collect, classify, and separate engorged female *Culex tritaeniorhynchus* mosquitoes to increase monitoring effectiveness [16]. In contrast, the virologic surveillance using chewable ropes to collect pig oronasal secretions can carry out at the beginning of transmission cycle (April, May and June in Taiwan) and widely apply to many pig farms. Additionally, this method is less technically demanding and caused less stress to pig than physically restraining pigs of collecting nasal swabs [13,23]. Oral fluid-has been used as the specimens for routine surveillance for other porcine respiratory diseases [41]. Thus oronasal secretions may be useful for routine JEV surveillance, especially for the epidemic area, such as Hokkaido, Japan, where it has been very challenge to identify circulating virus-positive mosquitoes [22]. However, mosquito surveillance is still critical in some area, such as Singapore, where pig farming has not been widely practiced [20].

Clustering of close related viruses in time and location identified by phylogenetic analysis supports the autochthonous circulation of virus in this study as well as in epidemic countries where mosquito numbers are low in cold months and JEV-positive mosquitoes are undetectable in the winter [4,16,17]. Experimentally infected pigs demonstrated JEV can persist in tonsil for 25 days even after seroconversion [13,23]. Persistent infection and oronasal transmission have been suspected to sustain JEV overwintering. However, we only observed oronasal transmission in May and June during a two-year surveillance in the operational pig farm. This mode of transmission might be less significant to support the overwintering of JEV GI in temperate regions. The operation model of commercial pig farming, all-in-all-out management practices, may prevent the persistence of virus in the pig population. The virus persistence in the tonsils of farm-raised, seropositive pigs should be further verified. In addition, a comprehensive ecological studies may require to determine the overwintering mechanism for JEV in epidemic countries.

We conclude pig’s oronasal secretion is an ideal specimen for genotype specific RT-PCR for virologic surveillance. It is a non-invasive and technically less demanding alternative to pig blood and mosquito pool for JEV molecular surveillance. Our conclusion is limited by insufficient sample size to estimate the virus positive rate in the blood samples and the small sample size of the oronasal secretions and mosquitoes collected from an operational pig farm. In the future, we plan to increase the number of collecting specimens from pig farms practicing a farrow-to-finish operation. The feasibility of using oronasal secretions for anti-JEV antibody detection (such as IgA) or JE vaccine evaluation require further study.

## Supporting information

S1 Table GI JEV strains detected in the pig farm during 2018–2019, Taiwan. (DOC)

S2 Table Neutralizing activity of JEV RT-PCR positive and negative pigs’ oronasal secretions against GI virus^*a*^. (DOC)

S1 Fig The rate of seroconversion obtained from 18 sentinel pigs in a pig rearing farm, Taiwan, 2009. (TIF)

S2 Fig Maximum-likelihood tree of JEV E gene detected in pig oronasal secretions (empty triangle) and mosquitoes (filled circle) in Taiwan, 2018–2019. (TIF)
