# Supplementary material for: The feasibility of field collected pig oronasal secretions as specimens for the virologic surveillance of Japanese encephalitis virus
Source: PLoS Negl Trop Dis. 2021 Dec 3;15(12):e0009977. doi: 10.1371/journal.pntd.0009977 (PMC8673640; doi:10.1371/journal.pntd.0009977)
Supplement: S1 Table — (DOC) [file pntd.0009977.s001.doc]

**S1 Table. GI JEV strains detected in the pig farm during 2018-2019, Taiwan, and the reference sequences isolated in Asia*^a^***

| Strain | Genotype/Subcluster | Source | Collection date | Accession no.*^b^* |
| --- | --- | --- | --- | --- |
| TC2018PigOS-1 | GI/II | Pig oronasal secretion | 2018 Jun 5 | MT075621 |
| TC2018PigOS-2 | GI/II | Pig oronasal secretion | 2018 Jun 5 | MT075622 |
| TC2018PigOS-3 | GI/I | Pig oronasal secretion | 2018 Jun 5 | MT075623 |
| TC2018-1 | GI/II | *Culex tritaeniorhynchus* | 2018 Jun 1 | MT075624 |
| TC2018-2 | GI/II | *Culex tritaeniorhynchus* | 2018 Jun 1 | MT075625 |
| TC2018-3 | GI/II | *Culex tritaeniorhynchus* | 2018 Jun 5 | MT075626 |
| TC2018-4 | GI/I | *Culex tritaeniorhynchus* | 2018 Jun 12 | MT075627 |
| TC2018-5 | GI/I | *Culex tritaeniorhynchus* | 2018 Nov 30 | MT075628 |
| TC2019PigOS-1 | GI/I | Pig oronasal secretion | 2019 May 24 | MT075618 |
| TC2019PigOS-2 | GI/I | Pig oronasal secretion | 2019 May 24 | MT075619 |
| TC2019PigOS-3 | GI/I | Pig oronasal secretion | 2019 Jun 5 | MT075620 |
| TC2019-1 | GI/I | *Culex tritaeniorhynchus* | 2019 Jun 7 | MT075629 |
| TC2019-2 | GI/I | *Culex tritaeniorhynchus* | 2019 Jun 17 | MT075630 |
| TC2019-3 | GI/I | *Culex tritaeniorhynchus* | 2019 Sep 27 | MT075631 |
| TC2009-2 | GI/I | Mosquitoes | 2009 | JF499792.1 |
| TC2010-1 | GI/I | Mosquitoes | 2010 | JF499810.1 |
| TC2009-1 | GI/I | Mosquitoes | 2009 | JF499791.1 |
| JEV/Taiwan/TC1106I/M/2011 | GI/I | Mosquitoes | 2011 | KF667299.1 |
| JEV/Taiwan/H10100739/H/2012 | GI/I | Human | 2012 | KF667324.1 |
| TC2010-2 | GI/I | Mosquitoes | 2010 | JF499811.1 |
| JaNAr17-07 | GI/I | Mosquitoes | 2007 | FJ185149.1 |
| LX29P-09 | GI/I | Human | 2009 | HM204529.1 |
| VNKT/486/2007 | GI/I | Mosquitoes | 2007 | AB728500.1 |
| VNKT/479/2007 | GI/I | Mosquitoes | 2007 | AB728499.1 |
| DH10M585 | GI/I | Mosquitoes | 2010 | KU295086.1 |
| GZDJ1609 | GI/II | Mosquitoes | 2016 | MF979779.1 |
| seal/china/anheal/2017 | GI/II | Seal | 2017 | MH165313.1 |
| SXYC1523 | GI/II | Mosquitoes | 2015 | KY078829.1 |
| ZJ-YW-437-18 | GI/II | Mosquitoes | 2018 | MK095916.1 |
| ZJ-YW-313-17 | GI/II | Mosquitoes | 2017 | MK095854.1 |
| ZJ-YW-305-17 | GI/II | Mosquitoes | 2017 | MK095852.1 |
| JEV/MQ/Yamaguchi/803/2016 | GI/II | Mosquitoes | 2016 | LC461956.1 |
| JEV/MQ/Yamaguchi/2013-1 | GI/II | Mosquitoes | 2013 | AB981183.1 |
| JEV/Bo/Miyazaki/1/2009 | GI/II | Bos taurus | 2009 | AB795032.1 |
| TPC0806c/M/2008 | GI/II | Mosquitoes | 2008 | KF667316.1 |
| JEV/Taiwan/TC1206a/M/2012 | GI/II | Mosquitoes | 2012 | KF667304.1 |
| YL2009-4 | GI/II | Mosquitoes | 2009 | JF499808.1 |
| TC2009-4 | GI/II | Mosquitoes | 2009 | JF499794.1 |
| JEV/Taiwan/TC1106i/M/2011 | GI/II | Mosquitoes | 2011 | KF667322.1 |
| TC2009-3 | GI/II | Mosquitoes | 2009 | JF499793.1 |
| JEV/Taiwan/TC1106n/M/2011 | GI/II | Mosquitoes | 2011 | KF667300.1 |
| TC2010-5 | GI/II | Mosquitoes | 2010 | JF499814.1 |
| K13BS124 | GI/II | Mosquitoes | 2013 | KM496496.1 |
| K10CT675 | GI/II | Mosquitoes | 2010 | JX018168.1 |
| JEV/sw/Okinawa/186/2014 | GI | Pig | 2014 | LC079039.1 |
| JEV/sw/Okinawa/153/2015 | GI | Pig | 2015 | LC075515.1 |
| JE KK 1116 | GI | NA | NA | DQ343290.1 |
| VNTN/04/2008 | GI | Mosquitoes | 2008 | AB728501.1 |
| JEV/sw/Thailand/185/2017 | GI | Pig | 2017 | LC461958.1 |
| KE-93-83 | GI | Mosquitoes | 1983 | KF192510.1 |
| JKT5441 | GII | Mosquitoes | 1980 | JQ429306.1 |
| JKT1754 | GII | Mosquitoes | 1979 | JQ429290.1 |
| SA14 | GIII | Mosquitoes | NA | AY243842.1 |
| CH1392 | GIII | Mosquitoes | 1990 | U44960.1 |
| JEV/sw/Mindanao/K3/2018 | GIII | Pig | 2018 | LC461959.1 |
| JKT7089 | GIV | Mosquitoes | 1981 | JQ429309.1 |
| JKT7180 | GIV | Mosquitoes | 1981 | JQ429310.1 |
| JEV/sw/Bali/93/2017 | GIV | Pig | 2017 | LC461961.1 |
| 10-1827 | GV | Mosquitoes | 2010 | JN587258.1 |
| Muar | GV | Human | 1954 | HM596272.1 |

*^a^*GI JEV, genotype I Japanese encephalitis virus; NA, not available.

*^b^*Full length of envelope gene.
