# Supplementary material for: The feasibility of field collected pig oronasal secretions as specimens for the virologic surveillance of Japanese encephalitis virus
Source: PLoS Negl Trop Dis. 2021 Dec 3;15(12):e0009977. doi: 10.1371/journal.pntd.0009977 (PMC8673640; doi:10.1371/journal.pntd.0009977)
Supplement: S2 Table — (DOC) [file pntd.0009977.s002.doc]

**S2 Table. Neutralizing activity of JEV RT-PCR positive and negative pigs’ oronasal secretions against GI virus*^a^***

| Collection time | Oronasal samples | FRμNT_50_ | FRμNT_70_ | RT-PCR |
| --- | --- | --- | --- | --- |
| June-2018 | 1 | <10 | <10 | + |
|  | 2 | NA | NA | - |
|  | 3 | 20 | 10 | - |
|  | 4 | <10 | <10 | - |
|  | 5 | NA | NA | + |
|  | 6 | 10 | <10 | - |
|  | 7 | <10 | <10 | + |
|  | 8 | NA | NA | - |
|  | 9 | 10 | <10 | - |
|  | 10 | 10 | <10 | - |
| May-2019 | 1 | <10 | <10 | - |
|  | 2 | <10 | <10 | - |
|  | 3 | 20 | 10 | - |
|  | 4 | <10 | <10 | - |
|  | 5 | <10 | <10 | + |
|  | 6 | 10 | <10 | - |
|  | 7 | 10 | 10 | + |
|  | 8 | <10 | <10 | - |
|  | 9 | 10 | <10 | - |
|  | 10 | 10 | 10 | - |

*^a^*Neutralizing antibody was measured by FRμNT (1). The FRμNT_50_ and FRμNT_70_ titers were obtained from reciprocal of the dilution of oronasal samples in reduction of foci-forming units by at least 50% and 70% relative to virus-only control, respectively. GI virus, genotype I TC2009-1 virus. NA, not available.

**Reference**

1. Fan YC, Chen JM, Lin JW, Chen YY, Wu GH, Su KH, et al. Genotype I of Japanese Encephalitis Virus Virus-like Particles Elicit Sterilizing Immunity against Genotype I and III Viral Challenge in Swine. Sci Rep. 2018;8(1):7481.
