# Supplementary figures and images for: The feasibility of field collected pig oronasal secretions as specimens for the virologic surveillance of Japanese encephalitis virus
Source: PLoS Negl Trop Dis. 2021 Dec 3;15(12):e0009977. doi: 10.1371/journal.pntd.0009977 (PMC8673640; doi:10.1371/journal.pntd.0009977)

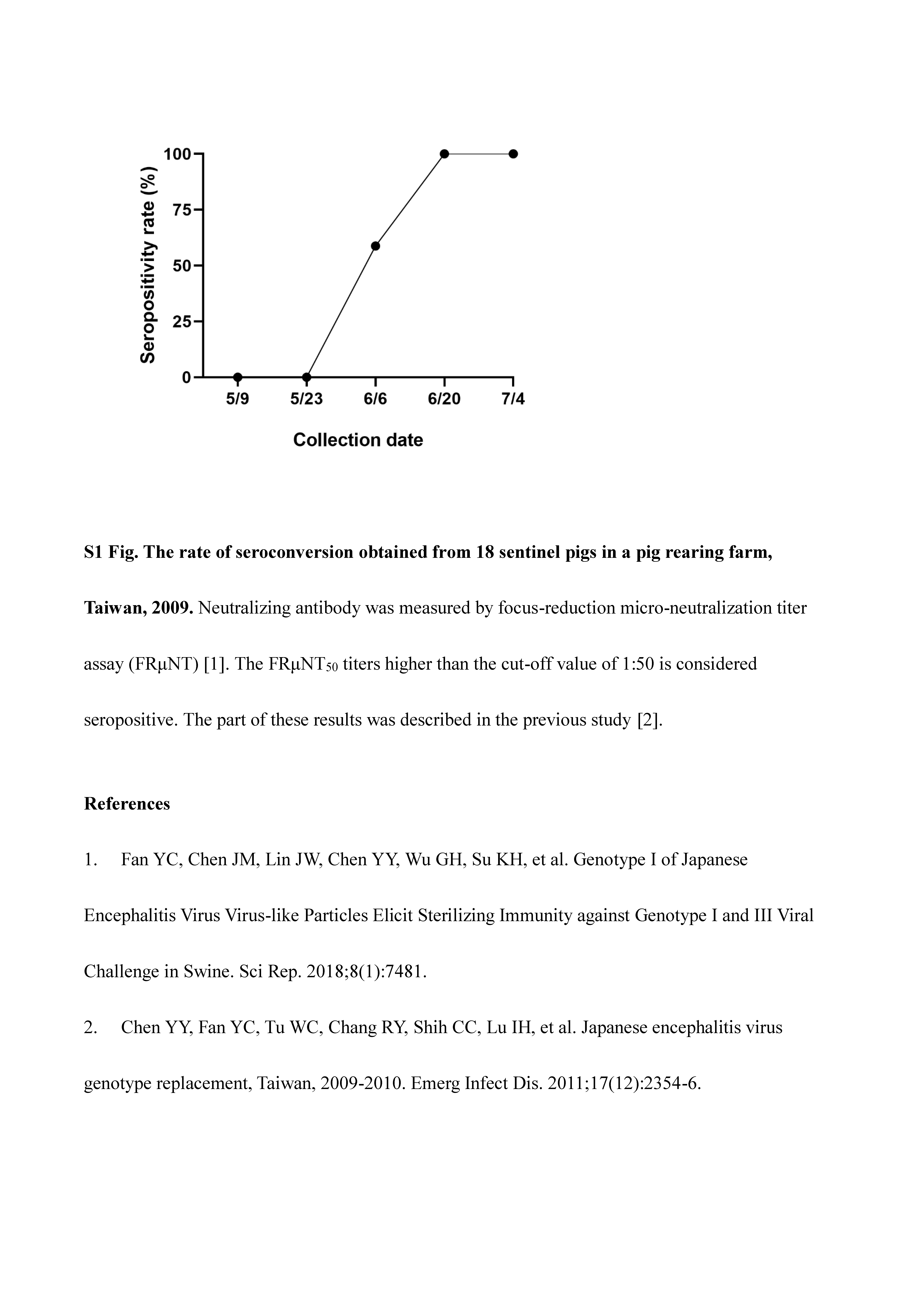

Supplement: S1 Fig — (TIF) [file pntd.0009977.s003.tif]

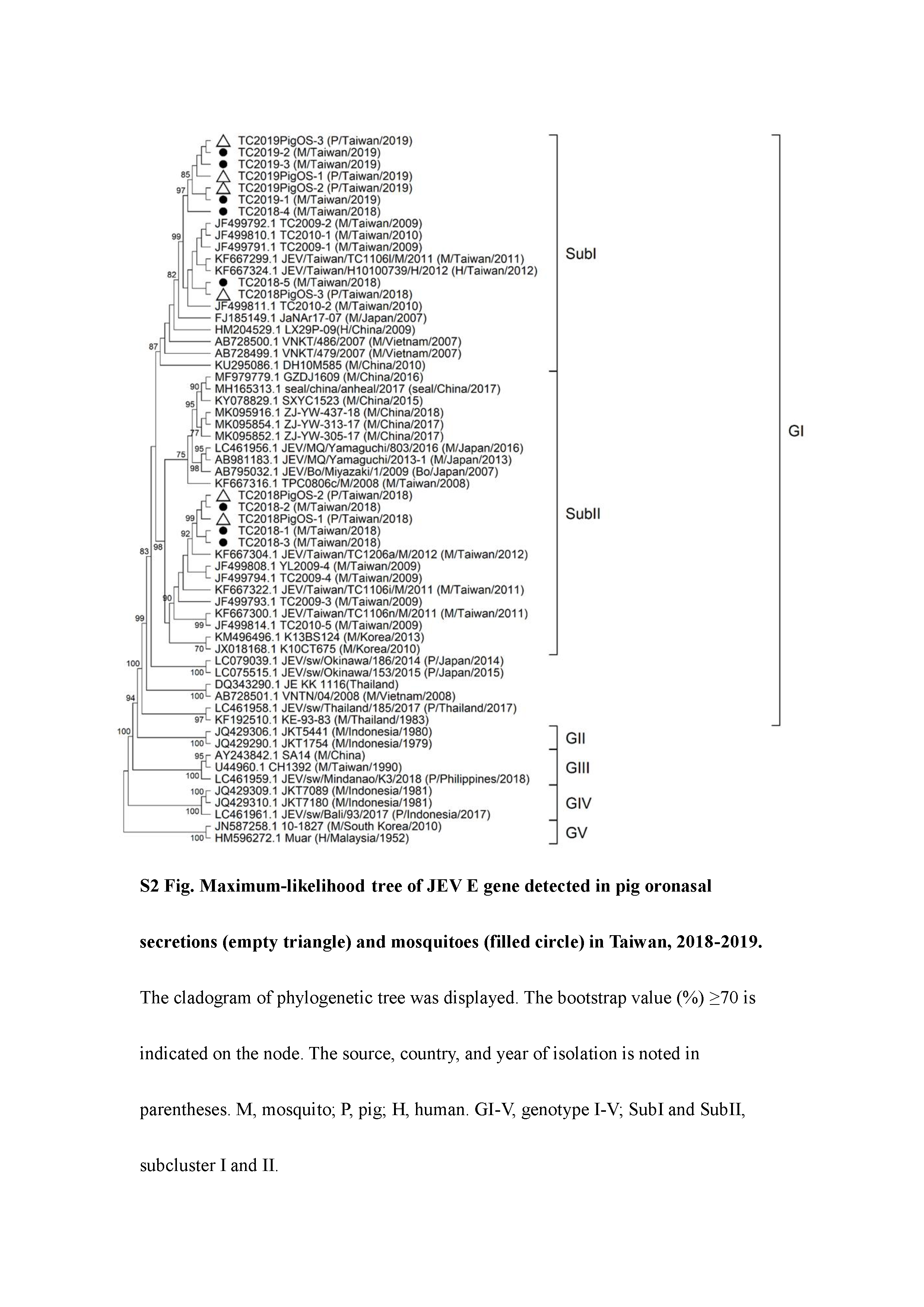

Supplement: S2 Fig — (TIF) [file pntd.0009977.s004.tif]
